# Microtranscriptome analysis of sugarcane cultivars in response to aluminum stress

**DOI:** 10.1371/journal.pone.0217806

**Published:** 2019-11-07

**Authors:** Renan Gonçalves da Silva, Thiago Mateus Rosa-Santos, Suzelei de Castro França, Pratibha Kottapalli, Kameswara Rao Kottapalli, Sonia Marli Zingaretti

**Affiliations:** 1 São Paulo State University (UNESP), School of Agricultural and Veterinarian Sciences, Jaboticabal, São Paulo, Brazil; 2 Department of Biotechnology, University of Ribeirão Preto, Ribeirão Preto, SP, Brazil; 3 Center for Biotechnology and Genomics, Texas Tech University, Lubbock, Texas, United States of America; ICAR-Indian Institute of Agricultural Biotechnology, INDIA

## Abstract

Although several metallic elements are required for plant growth, excessive amounts of aluminum ions (Al^3+^) can result in the inhibition of root growth, thus triggering water and nutrient deficiencies. Plants under stress undergo gene expression changes in specific genes or post-transcriptional gene regulators, such as miRNAs, that can lead to stress tolerance. In this study, we investigated the miRNAs involved in the response of sugarcane to aluminum stress. Four miRNA libraries were generated using sugarcane roots of one tolerant and one sensitive sugarcane cultivar grown under aluminum stress and used to identify the miRNAs involved in the sugarcane aluminum toxicity response. The contrast in field phenotypes of sugarcane cultivars in the field during aluminum stress was reflected in the micro-transcriptome expression profiles. We identified 394 differentially expressed miRNAs in both cultivars, 104 of which were tolerant cultivar-specific, 116 were sensitive cultivar-specific, and 87 of which were common among cultivars. In addition, 52% of differentially expressed miRNAs were upregulated in the tolerant cultivar while the majority of differentially expressed miRNAs in the sensitive cultivar were downregulated. Real-time quantitative polymerase chain reaction was used to validate the expression levels of differentially expressed miRNAs. We also attempted to identify target genes of miRNAs of interest. Our results show that selected differentially expressed miRNAs of aluminum-stressed sugarcane cultivars play roles in signaling, root development, and lateral root formation. These genes thus may be important for aluminum tolerance in sugarcane and could be used in breeding programs to develop tolerant cultivars.

## Introduction

Sugarcane (*Saccharum* spp.) is an important crop worldwide and a major source of sugar and ethanol. Brazil produces the world’s highest annual amount of sugarcane (740 Mt) followed by India (350 Mt), China, and Thailand [1]. Based on projected increases in worldwide demands for food and energy, global sugarcane production will increase by 21% by 2024 [2]. Production can be improved by increasing productivity and the amount of sugarcane cultivation area. The increase in cultivation area is evident in Brazil, where more than 9.5 million hectares are currently used for sugarcane cultivation, which is expected to increase to 10.5 million ha by 2023–24 due to the increasing demand for sugar and ethanol [3].

Among the main factors affecting agricultural productivity, soil is fundamentally important because it provides physical support, water, and nutrients for plant growth. Aluminum (Al), together with silicon and oxygen, are the three most abundant elements in the earth’s crust. Although certain metallic elements are required for plant growth, aluminum ions (Al^3+^) are a major abiotic factor affecting agricultural productivity [4]. Al is a nonessential element naturally found in the soil but it toxic and has high bioavailability in acidic soils of pH of 5.5 or lower, resulting in inhibition of root growth, architecture alteration, and elongation disruption [4]. Around the world, 50% of arable soils are acidic [5] while in Brazil acidic soil comprises 500 million hectares, with 70% of this land used for sugarcane cultivation [6].

Most Al^3+^ accumulates in root apoplast then translocates to other tissues [7], and the effects of Al^3+^ on roots and plant development depends on exposure time and aluminum concentration. The effects of Al^3+^ on plant metabolic processes can be observed just a few minutes after exposure. In plants exposed to 1.4 μM Al^3+^, after 30 min Al^3+^ was detected in the nuclei, thus inhibiting cell division and cell viability. Due to the rapid action of Al^3+^, the first Al^3+^-induced changes occur in the cell wall, plasma membrane, cytoskeleton, and the cell nucleus [8]. In roots, such changes inhibit root growth and they become shorter and thicker, absorbing less nutrients and water, and transport molecules more slowly through cells [9,10], triggering water stress and nutrient and mineral deficiencies [11]. In sugarcane, root growth inhibition can reach 46% under Al stress [12].

Tolerance and sensitivity under stress conditions are determined by plant genome composition, as well as through selective expression or post-transcriptional regulation of specific genes. Such regulation can be achieved through the expression of transcription factors (TFs) like MYB, a key player in regulating plant responses to abiotic stress [13], or by small noncoding RNAs called microRNAs (miRNAs). miRNAs are 20 to 24 nucleotide-long single-stranded RNA sequences that play roles in post-transcriptional gene silencing (PTGS) in plants [14–16]. The first identified miRNAs were reported to help modulate physiological and biochemical processes involved plant development and adaptation [17]. Since then, miRNAs have been identified in a variety of plant species, including *Arabidopsis thaliana* [18], *Triticum aestivum* L. [19], *Glycine max* [20], and *Manihot esculenta* [21], suggesting that miRNAs play important roles in the regulation of molecular responses to biotic and abiotic stress.

Over the last years, miRNAs have been intensively studied, yet not much is known about plant responses to metallic element stress, especially those of crop plants. Currently available information about aluminum stress responses in plants comes from studies of model plants like *Medicago truncatula* [22,23] and *Arabidopsis thaliana* [24,25]. During metal exposure stress, gene expression can be modified to regulate different compensatory mechanisms such complexing metals with ligands such as glutathione, phytochelatins, and metallothioneins, repression of oxidative stress, and signal transduction for different biological process [26,27]. Some miRNAs, such as miR159, miR160, miR319, and miR396, are downregulated in *Medicago truncatula* seedling roots after 4 hours of aluminum stress, and their targets are transcription factors related to seed germination, embryo development, and cold and drought responses [23].

In sugarcane, several miRNAs associated with cold [28] and drought [29–31] tolerance have been identified, however, there is no information about involvement of miRNAs in response to Al stress. Our goal was to identify the miRNAs involved in responses of multiple sugarcane cultivars to aluminum stress. In this study, we focused on differential miRNA expression analysis and quantitative real-time PCR (RT-qPCR) validation in sugarcane roots exposed to increased levels of aluminum (Al^3+^).

## Materials and methods

### Plant materials and RNA isolation

Pre-germinated plants from the sugarcane (*Saccharum* spp) cultivars, CTC-2, also called tolerant to aluminum stress (TAS), and RB-855453, also called sensitive to aluminum stress (SAS), were grown using a hydroponic system in a greenhouse at 26°C to 30°C and with 8/16 h dark/light cycles. For 30 d, the plants were kept in 16 L containers filled with standard hydroponic solution [32] before cultivation for 7 d with the addition of either 0.0 or 221 μmol Al^3+^ L^-1^ at pH 4.5. After 7 d, roots were collected and immediately frozen in liquid nitrogen and stored at -80°C for further use. Total RNA was isolated from root samples from control and stressed plants using the Sigma plant RNA kit (Sigma, Inc, USA). RNA quality and concentration were determined using a Qubit 2.0 fluorometer (Life Technologies, USA).

### miRNA library and sequencing

cDNA libraries were generated using Illumina True-Seq small RNA prep (Illumina, USA) and sequenced using 35 bp single end sequencing on a MiSeq sequencer (Illumina, Inc, USA) following the manufacturer’s instructions.

### Real time PCR of miRNAs

In order to validate our miRNA transcriptome, we performed RT-qPCR analysis of randomly selected miRNAs using quantitative real time polymerase chain reaction (RT-qPCR) [33]. The RevertAid First Strand cDNA Synthesis kit (Thermo Fisher Scientific, USA) was used for cDNA synthesis following the manufacturer’s instructions. For RT-qPCR experiments, cDNA concentrations were standardized for each sample and dissociation curve analysis performed to check primer specificity. Reactions were performed with a total reaction volume of 20 μL containing 1 μg RNA, DNAse treated, 200 U of RevertAid M-MuLV Reverse Transcriptase, 20 mM dNTPs, 20 U RiboLock RNase Inhibitor, 5X reaction buffer (Thermo Fisher Scientific, USA), 1 μM RT specific Primer, and 100 μM dT primer, which were mixed then incubated at 42°C for 60 min and 5 min at 70°C. Real-time PCR was carried out in a Stratagene MX3005P thermocycler using SYBR Green Jump Start Taq Ready Mix (Sigma Aldrich, USA) for quantification. Thermal cycling conditions were 94°C for 2 min followed by 40 cycles of 94°C for 15 s, 60°C for 1 min, and 72°C for 30 s.

The miRNA levels were quantified after normalization to 18S rRNA gene levels as an internal control. Gene-specific primers used in real time experiments and miRNA sequences are shown in S2 and S3 Tables. For validation, root samples collected after 7 d of aluminum stress (DAS) were used and miRNA expression levels analyzed using MxPro QPCR software 4.10 version (Stratagene, USA). Three biological replicates were tested to ensure reproducibility.

### miRNA target prediction and functional annotation

The miRNAs targets were predicted using Mercator (http://mapman.gabipd.org/web/guest/app/Mercator) to search for targets genes based on MapMan "BIN" ontology, which is tailored for the functional annotation of plant "omics" data [34]. The GO (Gene Ontology) categorization was performed using three independent hierarchies for biological process, cellular component, and molecular function using the UniProt Knowledgebase (https://www.uniprot.org) and QuickGO (EMBL-EBI, https://www.ebi.ac.uk/QuickGO) tools. The data from each individual biological library was deposited to the NCBI SRA database with the SRA accession IDs: SRR9035251, SRR9035250, SRR9035245, SRR9035244, SRR9035249, SRR9035248, SRR9035243, SRR9035242, SRR9035253, SRR9035252, SRR9035247, and SRR9035246.

## Results

### Construction and sequencing analysis of miRNAs library

**Fig 1:**
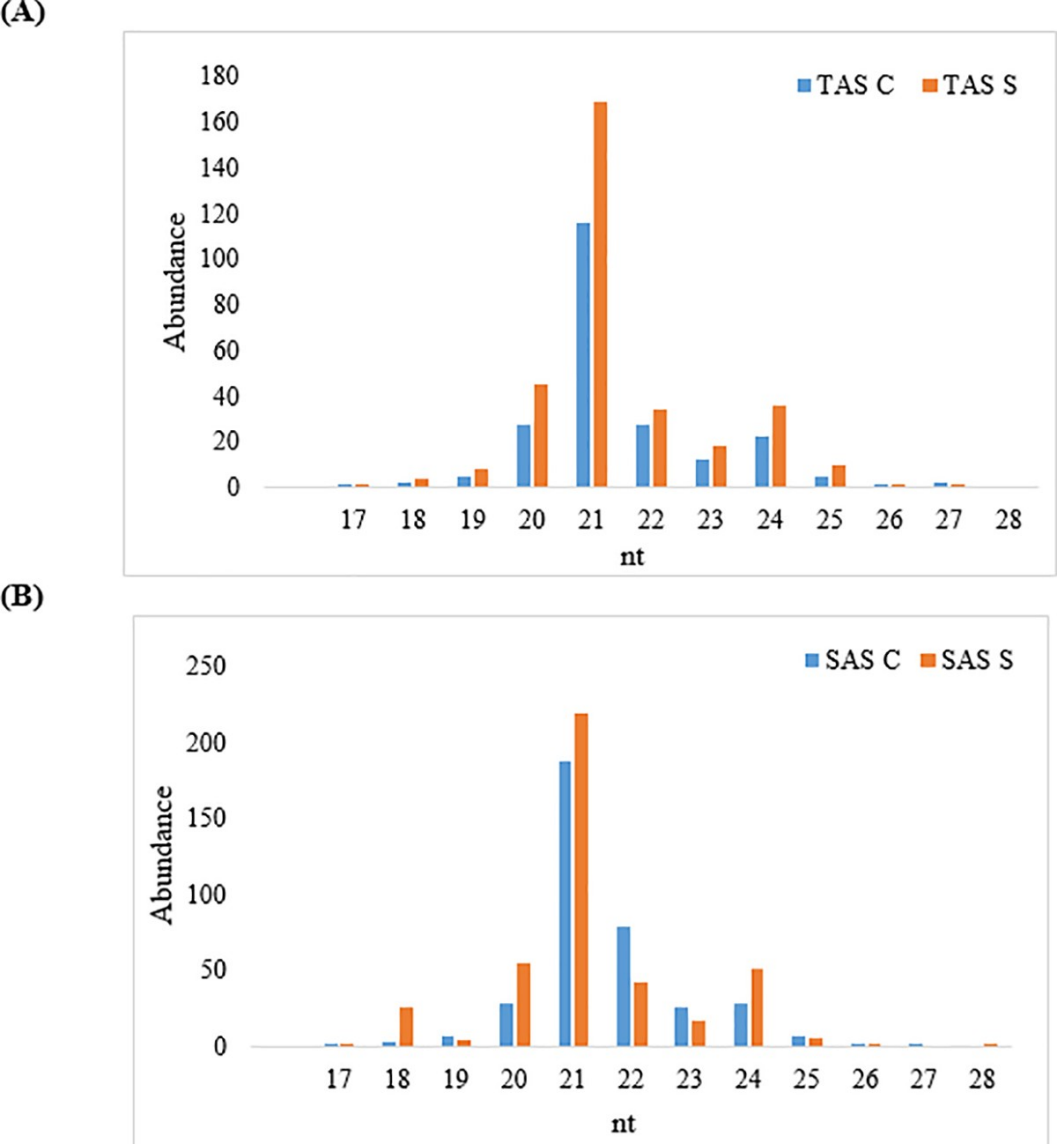
Size distributions of miRNA sequences in two sugarcane cultivars. (A) Abundance of miRNA sizes in tolerant cultivar. TAS C–Tolerant aluminum stress control; TAS S–Tolerant aluminum stress stressed. (B) Abundance of miRNA sizes in sensitive cultivar. SAS C–Sensitive aluminum stress control; SAS S–Sensitive aluminum stress stressed.

To identify miRNAs involved in aluminum stress responses, four miRNA libraries were generated from the sugarcane roots of the sugarcane cultivars CTC-2 (Tolerant Aluminum Stress, TAS) and RB-855453 (Sensitive Aluminum Stress, SAS) that were exposed to aluminum stress for 7 d. These miRNA libraries were then sequenced using Illumina technology. Over 12 million raw reads, with a Q-Score of 37 and 53% CG content, were obtained. After processing and filtering for poor quality sequences, 5.8 million clean sequences from CTC-2 (TAS) and 6.2 million reads from RB-855453 (SAS) samples remained. About 20K reads were then assembled, 11.5 K from RB-855453 (SAS) and 8.5 K from CTC-2 (TAS). The size distribution of miRNAs ranged from 17 to 28 nt (Fig 1) and the majority of reads were 20 to 24 nt long, with 21 nt long miRNAs being the most abundant species for both cultivars. The size distributions of sugarcane root miRNAs are thus consistent with results observed for other plants using deep-sequencing approaches [35,36].

**Fig 2:**
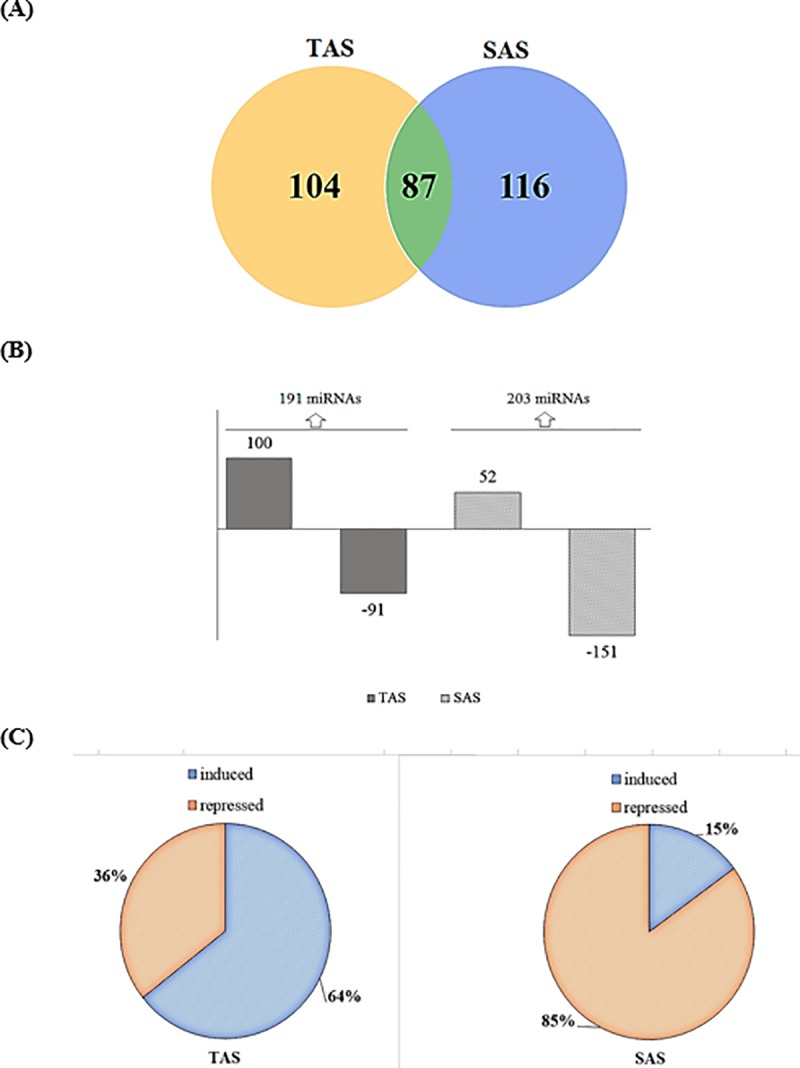
miRNAs expression profile. (A) Venn diagram showing miRNAs differently expressed in both cultivars; (B) The number of stress responsive miRNAs for each cultivar and the number of induced and repressed miRNAs under stress conditions; (C) Expression levels of common differentially expressed miRNAs between cultivars.

**Fig 3:**
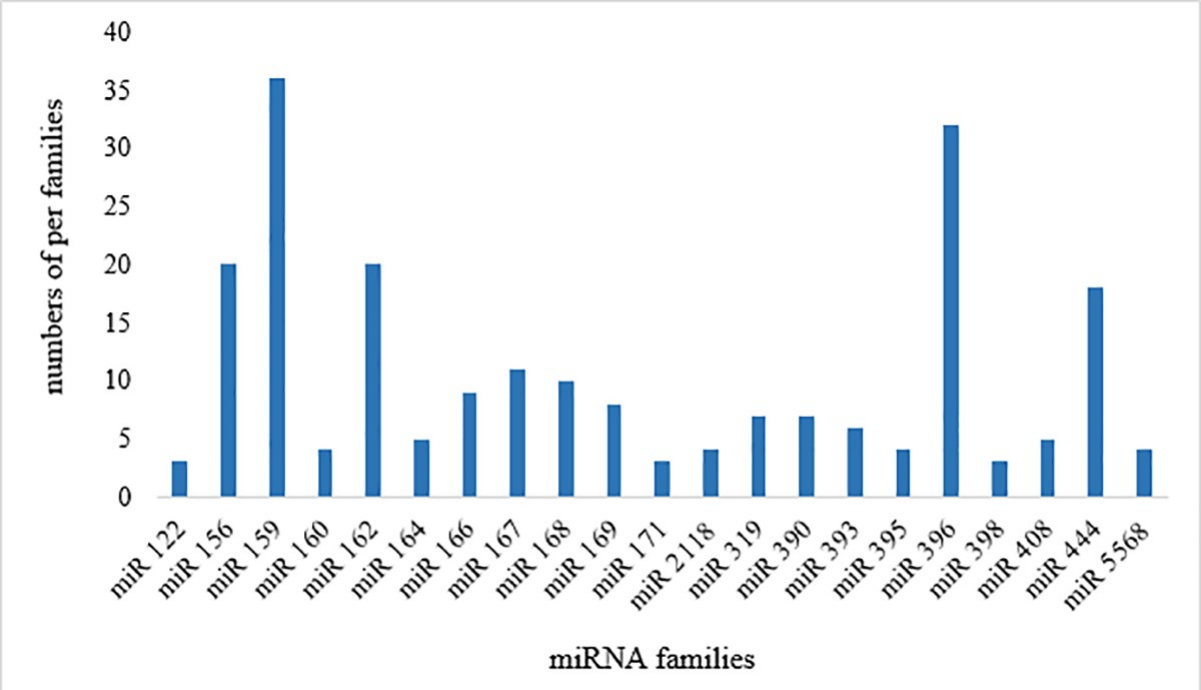
Most abundant miRNA families identified in sugarcane roots.

To identify the miRNAs involved in sugarcane responses to aluminum stress, we selected the differentially expressed miRNAs in both cultivars for further analysis. A total of 394 differentially expressed miRNAs were identified (S1 Table). For aluminum stresses samples, 104 differentially expressed miRNAs were specific to TAS while 116 were specific to SAS and 87 were in common for both cultivars (Fig 2A). In the TAS cultivar, out of a total of 191 differentially expressed miRNAs, 52% were upregulated while 75% of miRNA from the SAS cultivar were downregulated (Fig 2B). The aluminum sensitive and tolerant cultivars thus had opposing miRNA expression profiles. For the TAS cultivar, 64% of miRNAs were induced while 85% of miRNAs were repressed in the SAS cultivar (Fig 2C). Generally, plant miRNAs can be classified into several different families whose members have similar sequences. The miRNAs identified in sugarcane roots belong to 100 known families (S1 Fig), with the most abundant miRNA families being miRNA159, miRNA156, miRNA 162, miRNA 396, and miRNA 444 (Fig 3).

Out of the 14 miRNAs families downregulated in the tolerant cultivar (TAS), nine of these families were also downregulated in the sensitive cultivar (SAS): miR156, miR159, miR164, miR166, miR169, miR393, miR398, miR444, and miR5568. Five families were thus downregulated only in the tolerant cultivar: miR121, miR122, miR408, miR2128, and miR6253) and two miRNAs families were upregulated in TAS (miR168 and miR395), and the miR395 family was upregulated in SAS (Table 1).

**Table 1:** Expression analysis (Log2FC) of miRNAs identified from sugarcane sequencing.

miRNA family		miRNA reference	Log2FC^1^	
			*TAS*	*SAS*
Downregulated	miR121	miR121-1-npr (sit)	-4,88	NR
	miR122	miR122-2-npr (sit)	-5,24	NR
	miR156	miR156a-4 (sit)	-2,95	-1,19
	miR159	miR159a (sbi)	-2,95	-1,01
	miR164	miR164c (sit)	NR	-2,17
		miR164f-3p (zma)	-1,36	NR
	miR166	miR166a-5p (zma)	-1,36	-2,19
	miR169	miR169n-5p (zma)	-1,36	-1,19
	miR393	miR393h (gma)	NR	-1,13
		miR393c-5p (zma)	-1,30	NR
	miR398	miR398b-5p (zma)	-1,36	-1,19
	miR408	miR408 (csi)	-1,36	NR
	miR444	miR444f (osa)	-2,36	-1,59
	miR2128	miR2128a-3p (gma)	-2,36	NR
	miR5568	miR5568g-3p (sbi)	NR	-2,78
		miR5568f-3p (sbi)	-1,36	NR
	miR6253	miR6253 (osa)	-2,30	NR
Upregulated	miR168	miR168a-5p (zma)	3,05	NR
	miR395	miR395a (sly)	4,85	1,12
Contrasting	miR160	miR160e-5p (osa)	-2,36	1,18
	miR162	miR162b (ptc)	NR	1,18
		miR162b (gma)	-1,36	NR
	miR167	miR167h-3p (osa)	4,29	-4,36
	miR171	miR171i (mdm)	3,34	-2,78
	miR319	miR319-2 (sit)	2,14	-2,01
	miR390	miR390a (cpa)	NR	1,18
		miR390a (ath)	-1,36	NR
	miR396	miR396d (zma)	3,27	-4,30

^1^NR: not responsive.

Contrasting expression changes were observed for seven miRNA families between TAS and SAS cultivars. In the TAS cultivar, miR160, miR162, and miR390 were downregulated while miR167, miR171, miR319, and miR396 were upregulated, with the opposite expression profiles observed in the SAS cultivar (Table 1). As shown in Table 1, the expression of some miRNAs was not detected in sequencing; therefore, these miRNAs were considered not responsive (NR), although the related miRNA families could be classified as upregulated or downregulated.

### miRNA transcriptome validation by RT-qPCR

**Fig 4:**
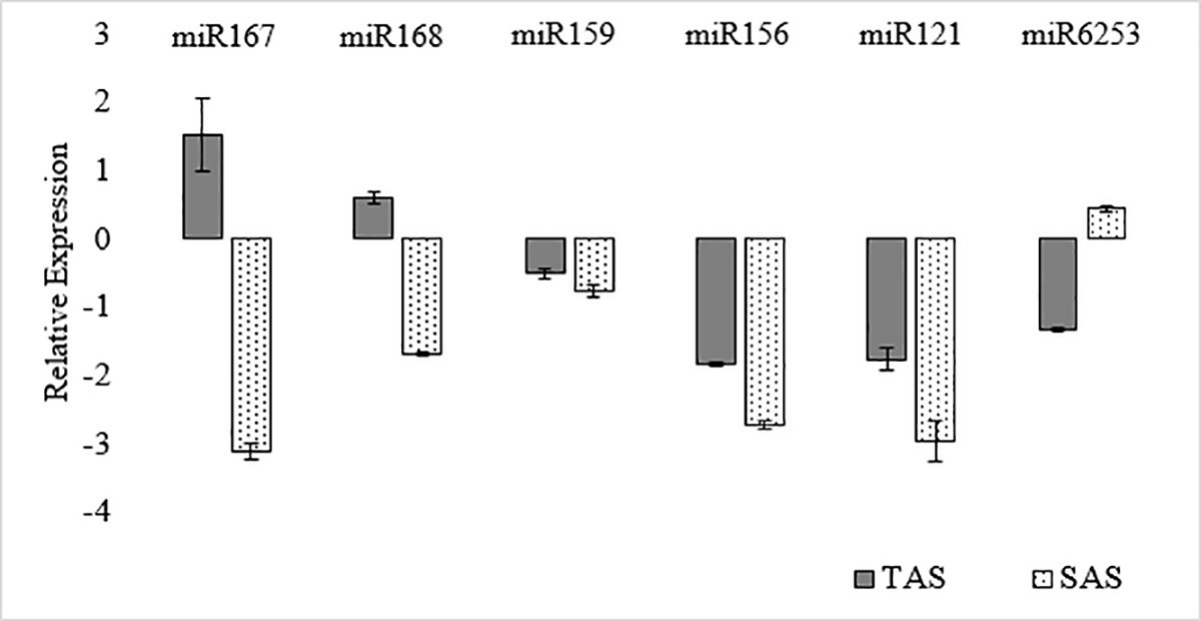
Relative expression of six identified miRNAs in sugarcane. Tolerant cultivar (TAS) and sensitive cultivar (SAS).

From the 394 differentially expressed miRNAs modulated by aluminum stress (S1 Table), 6 miRNAs (miR167, miR168, miR6253, miR159, miR156, and miR121) were randomly selected based on the highest and lowest levels of expression in the TAS cultivar. Sequencing results for all these miRNAs were confirmed by RT-qPCR and results were consistent with those from high-throughput sequencing analyses (Fig 4).

### Prediction of miRNA targets and GO annotation

Because plant miRNA sequences are highly complementary to their targets, these sequences can be used for target predictions [37]. To better understand the possible biological functions of differentially expressed miRNAs during aluminum stress responses, the target of miRNAs from the most abundant microRNA families identified were searched for using Mercator, which assigns functional terms to nucleotide sequences (Table 2; S4 Table). The functional annotation of targets is shown in S4 Table. The genes and transcription factors regulated by the miRNAs identified participate in several biological processes, including cell growth regulation (*LRR protein*), auxin-activated signaling pathways (*Auxin response factor*), osmotic stress responses (*CBL-interacting protein kinase 1*), and negative regulation of growth (*MYB domain protein 33*), among others.

**Table 2:** Predicted miRNA targets.

miRNA	Potential targets identified by Mercator
121	*K*^*+*^ *uptake permease*
156	*Squamosa promoter-binding protein-like*
159	*MYB domain protein; LRR protein*
160	*Auxin response factor*
164	*NAC domain containing protein*
167	*OsWAK; Copper-transporting ATPase PAA1*
169	*12-oxo-phytodienoic acid reductase*
319	*MYB domain protein*
396	*Growth-regulating factor*
398	*Copper/zinc superoxide dismutase*
444	*MADS-box transcription factor*

## Discussion

Due to their regulatory roles during plant development, the study of microRNAs associated with biotic and abiotic stress has dramatically increased. Several miRNAs have been identified in sugarcane in different tissues and stress conditions [29,38], but none have been reported for sugarcane under aluminum stress. Here we report the first microtranscriptome analysis associated with aluminum stress responses in sugarcane. The contrasting response of sensitive and tolerant cultivars in the field was reflected in the opposing microtranscriptome profiles obtained. During aluminum stress, while 64% of microRNAs were induced in the tolerant cultivar, in the sensitive cultivar, 85% of microRNAs were repressed under aluminum stress (Fig 2C). Six of these miRNAs were confirmed to have comparable expression profiles based on sequencing and RT-qPCR results (Fig 4).

Differentially expressed miRNAs were classified into 100 different families (S1 Fig), the most abundant of which were miRNA159, miRNA156, miRNA 162, miRNA 396, and miRNA 444 (Fig 3). Members of those miRNA families have been identified during different stress conditions in several crops [27]. Among the differentially expressed miRNA families, we selected miR121, miR159, miR160, miR164, miR393, and miR398, for further analysis, as these families (except miR121) were differentially expressed in both TAS and SAS during aluminum stress (Table 1 and S1 Table).

**Fig 5:**
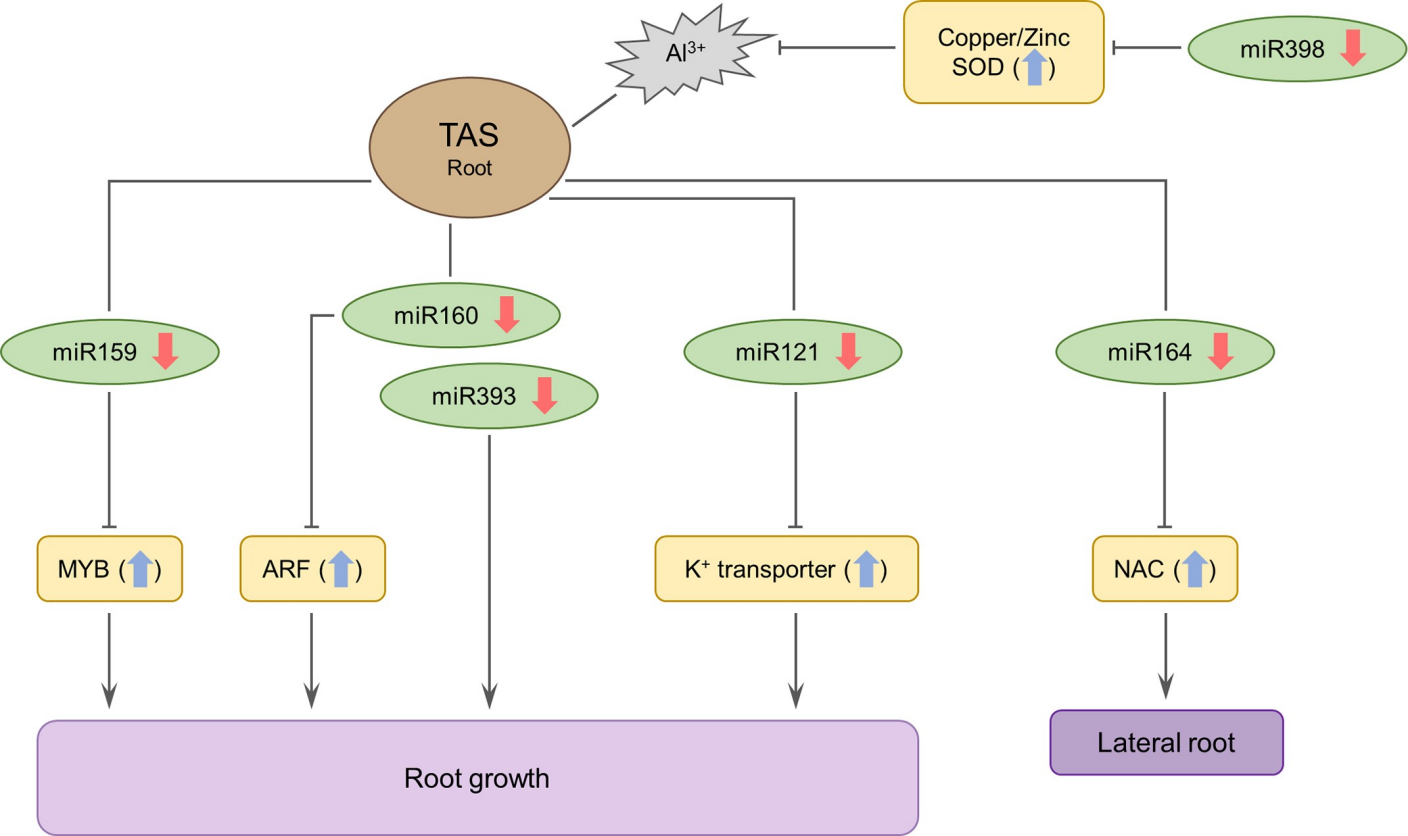
A model for aluminum stress responses in TAS.

In our study, we showed that the differentially expressed miRNAs likely modulate target genes involved in signaling, root development, and lateral root formation, which may explain the tolerance mechanism of the TAS cultivar (Fig 5). Reactive oxygen species (ROS) production is increased by several environmental stresses, including exposure to drought and heavy metals [39]. In addition, recent studies indicate that SODs as one of the primary types of antioxidant enzymes and are responsible for maintaining ROS gradients to guide plant developmental processes [40]. miR398 is predicted to regulate *copper/zinc superoxide dismutase* (Table 2), an isoform of the oxidative stress-response enzyme *SOD* (*superoxide dismutase*) (S4 Table). Downregulation of miR398 in TAS will increase of *SOD* expression, promoting the downregulation of 5 additional miRNAs (miR159, miR160, miR393, miR121, and miR164).

Both miR160 and miR393 participate in the auxin signaling pathway by regulating the target genes *auxin response factor* (*ARF*) and *transport inhibitor response 1* (*TIR1*), respectively, which are required for normal auxin responses and are essential for many important biological process in plants [41,42], including root development [43]. In addition, the miR393 family was downregulated in TAS and SAS while the miR160 family was downregulated in TAS and upregulated in SAS during aluminum stress (Table 1). The repressed expression of miR160 in the TAS cultivar will increase *ARF* levels, leading to the inhibition of cell wall modification and promotion of root growth [44]. According to the authors [44], the auxin signaling pathway may also be a strategy for plant detoxification.

Downregulation of miR398 reduces oxidative stress caused by ROS while repression of miR159, miR160, miR393, miR121, and miR164 modulate signaling, root development, and lateral root formation.

The miR159 and miR164 families also participate in biological signaling processes mediated by plant phytohormones (S4 Table) and regulate the target genes *MYB domain protein* and *NAC domain containing protein*, respectively (Table 2). miR159 has also been associated with aluminum stress in rice [45] and represses primary root growth through modulation of root meristem size. In our study, miR159 was repressed in TAS, indicating that this miRNA positively influences the root growth by reducing modulation of the *MYB* target gene involved in cell cycle progression [46].

One of the first symptoms of Al^3+^ toxicity in plants is the reduction of lateral root formation [47,48]. Our results showed repression of miR164 in both cultivars during aluminum stress, which is expected to increase *NAC* expression and thus promote lateral root formation and the expansion of the completely radicular system, thus increasing water and nutrient uptake.

miR121 was one of the most repressed miRNAS in TAS, with a −4.88-fold reduction and was not responsive in SAS (Table 1). The repression of miR121 likely results in increased levels of the membrane protein *K*^*+*^ *uptake permease* synthesis in TAS, promoting potassium transport and auxin distribution in roots [49].

## Conclusions

Specific miRNAs that are differentially expressed in TAS and SAS play roles in signaling, root development, and lateral root formation. This study represents one step towards understanding mechanisms underlying aluminum tolerance of the TAS cultivar.

## Supporting information

S1 Fig Summary of identified miRNA families in sugarcane and number of miRNAs per family. (TIF)

S1 Table Stress-responsive miRNAs identified in TAS and SAS. (DOCX)

S2 Table The primer sequences used in the RT-qPCR validation. (DOCX)

S3 Table miRNAs sequences evaluated. (DOCX)

S4 Table Distribution of predicted miRNA targets genes. Functional annotation of target genes regulated by the most abundant miRNA families differentially expressed.
(DOCX)
