# Supplementary figures and images for: Microtranscriptome analysis of sugarcane cultivars in response to aluminum stress
Source: PLoS One. 2019 Nov 7;14(11):e0217806. doi: 10.1371/journal.pone.0217806 (PMC6837492; doi:10.1371/journal.pone.0217806)

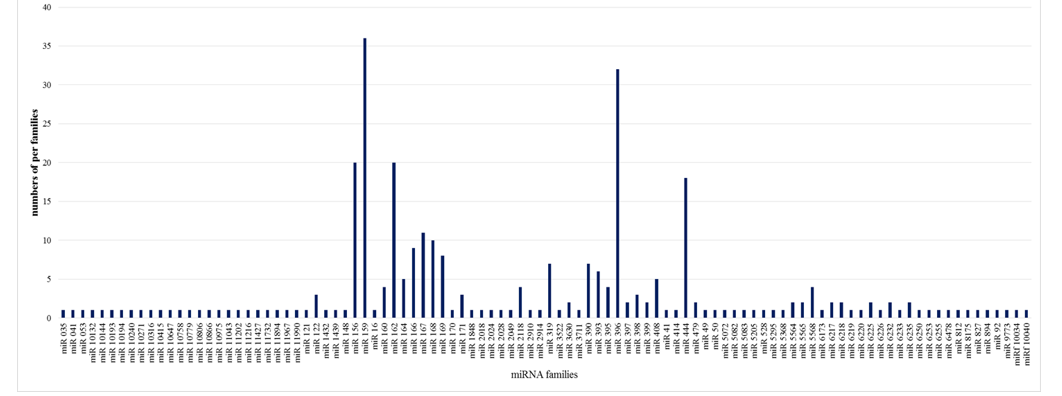

Supplement: S1 Fig — (TIF) [file pone.0217806.s001.tif]
