# Supplementary material for: Microtranscriptome analysis of sugarcane cultivars in response to aluminum stress
Source: PLoS One. 2019 Nov 7;14(11):e0217806. doi: 10.1371/journal.pone.0217806 (PMC6837492; doi:10.1371/journal.pone.0217806)
Supplement: S1 Table — (DOCX) [file pone.0217806.s002.docx]

**S1 Table**. **Stress-responsive miRNAs identified in TAS and SAS**.

**miRNA RB85 – FC^1^ RB85 - log2FC CTC – FC^1^ CTC - log2FC**

aly-miR159a-3p # # 0,39 -1,36

aly-miR159b-3p 0,44 -1,19 3,93 1,97

ath-miR159b 4,55 2,18 0,39 -1,36

ath-miR162b 0,44 -1,19 # #

ath-miR167d 2,17 1,12 # #

ath-miR390a # # 0,39 -1,36

ath-miR8175 0,33 -1,59 15,70 3,97

ath-miRf10316-npr # # 0,37 -1,43

bcy-miR396a # # 10,10 3,34

bdi-miR156b-3p 2,17 1,12 # #

bdi-miR159b-3p.1 # # 7,85 2,97

bdi-miR159b-5p.1 0,22 -2,19 0,39 -1,36

bdi-miR159c 2,27 1,18 # #

bdi-miR166c-5p 0,22 -2,19 # #

bdi-miR168-5p # # 3,62 1,86

bdi-miR169k-5p # # 9,64 3,27

bdi-miR319b-3p # # 4,36 2,13

bdi-miR396a-5p 0,46 -1,12 8,64 3,11

bdi-miR396b-5p 0,12 -3,01 6,54 2,71

bdi-miR396c-3p 2,27 1,18 # #

bdi-miR396d-3p 0,44 -1,19 # #

bdi-miR396d-5p # # 10,10 3,34

bdi-miR444b 2,27 1,18 # #

bdi-miR444c 0,44 -1,18 3,93 1,97

bdi-miR444d 0,25 -2,01 3,93 1,97

bna-miR156a # # 0,39 -1,36

bna-miR159 [3] 4,55 2,18 0,39 -1,36

bra-miR162-3p 0,44 -1,19 # #

bra-miR408-5p 0,22 -2,19 # #

cca-miR167 0,46 -1,13 # #

cme-miR159a # # 10,10 3,34

cme-miR162 0,22 -2,19 # #

cme-miR396b 2,27 1,18 # #

cme-miR396e 0,15 -2,78 20,19 4,34

cpa-miR390a 2,27 1,18 # #

csi-miR162 2,27 1,18 # #

csi-miR166e 0,22 -2,19 0,39 -1,36

csi-miR393 # # 9,64 3,27

csi-miR396a 0,44 -1,19 0,39 -1,36

csi-miR408 # # 0,39 -1,36

csi-miR479 2,17 1,12 # #

dpr-miR408 0,44 -1,19 # #

far-miR159 # # 5,23 2,39

far-miR396 0,38 -1,39 5,89 2,56

gba-miR162a 2,27 1,18 # #

gba-miR396l # # 0,39 -1,36

gba-miR396p 0,44 -1,19 # #

gba-miR396u 2,27 1,18 # #

ghr-miR162c 0,44 -1,19 # #

ghr-miR166i 4,55 2,18 # #

ghr-miR167a 2,17 1,12 # #

ghr-miR390b # # 0,39 -1,36

ghr-miR390d 2,27 1,18 # #

ghr-miR5368 0,48 -1,06 # #

gma-miR156q # # 3,05 1,61

gma-miR156s 2,98 1,58 # #

gma-miR159a-3p 2,27 1,18 # #

gma-miR162b # # 0,39 -1,36

gma-miR162c # # 0,39 -1,36

gma-miR167h # # 8,83 3,14

gma-miR2118a-3p # # 0,19 -2,36

gma-miR2118b-3p # # 0,39 -1,36

gma-miR390e # # 10,60 3,41

gma-miR393h 0,46 -1,13 # #

gma-miR393k 0,46 -1,13 # #

gma-miR396h 0,42 -1,26 # #

gso-miR3522a # # 0,39 -1,36

han-miR3630-3p 0,19 -2,42 2,36 1,24

han-miR3630-3p # # 2,36 1,24

hbr-miR159a 2,27 1,18 # #

hbr-miR396b 0,44 -1,19 # #

hbr-miR6173 4,77 2,25 10,60 3,41

hpe-miR162a 2,27 1,18 # #

htu-miR162a 0,44 -1,19 # #

htu-miR393c 2,17 1,12 # #

hvu-miR156 # # 0,39 -1,36

hvu-miR159a 0,33 -1,59 15,70 3,97

hvu-miR168-3p # # 8,83 3,14

hvu-miR168-5p 0,36 -1,49 6,34 2,66

hvu-miR444b 0,33 -1,59 3,93 1,97

lus-miR159c # # 7,85 2,97

lus-miR162b # # 0,39 -1,36

mdm-miR162b 2,27 1,18 # #

mdm-miR169b 0,46 -1,13 # #

mdm-miR171i 0,15 -2,78 10,10 3,34

mdm-miR396g 0,15 -2,78 30,29 4,92

mes-miR162 0,44 -1,19 # #

mes-miR166i 2,27 1,18 # #

mes-miR396a 0,44 -1,19 # #

mtr-miR162 2,27 1,18 # #

mtr-miR5205b # # 0,44 -1,17

mtr-miR5295b [2] # # 0,44 -1,17

nta-miR162b 0,44 -1,19 # #

oni-miR812 0,48 -1,06 0,21 -2,23

oru-miR159 # # 7,85 2,97

oru-miR396 0,44 -1,18 7,85 2,97

osa-miR1439 0,44 -1,19 # #

osa-miR156f-3p 0,46 -1,13 # #

osa-miR156g-3p 0,46 -1,13 # #

osa-miR156l-5p # # 0,39 -1,36

osa-miR159a.1 # # 10,10 3,34

osa-miR159b 0,33 -1,59 4,91 2,29

osa-miR159d 0,33 -1,59 0,39 -1,36

osa-miR160a-3p # # 10,10 3,34

osa-miR160e-5p 2,27 1,18 0,19 -2,36

osa-miR166a-5p 0,11 -3,19 # #

osa-miR166e-5p 0,22 -2,19 # #

osa-miR167e-3p 0,11 -3,19 10,10 3,34

osa-miR167h-3p 0,05 -4,36 19,63 4,29

osa-miR167i-3p # # 10,10 3,34

osa-miR168a-3p 0,50 -1,01 8,83 3,14

osa-miR168a-5p # # 5,67 2,50

osa-miR169o 0,44 -1,19 # #

osa-miR171f-5p 0,44 -1,19 # #

osa-miR171g 0,44 -1,19 # #

osa-miR1848 4,55 2,18 # #

osa-miR2118b 0,46 -1,13 # #

osa-miR2118n 0,23 -2,13 # #

osa-miR319a-3p.2-3p 0,42 -1,26 # #

osa-miR393b-3p 0,44 -1,19 # #

osa-miR396e-3p # # 8,83 3,14

osa-miR396e-5p 0,36 -1,48 3,14 1,65

osa-miR396f-5p 0,46 -1,13 # #

osa-miR399d # # 0,39 -1,36

osa-miR408-5p 0,44 -1,19 # #

osa-miR444b.2 0,10 -3,33 3,93 1,97

osa-miR444c.2 # # 0,39 -1,36

osa-miR444d.2 0,44 -1,19 0,39 -1,36

osa-miR444d.2 # # 0,39 -1,36

osa-miR444d.3 0,14 -2,82 # #

osa-miR444f 0,33 -1,59 0,19 -2,36

osa-miR5072 # # 0,41 -1,30

osa-miR5082 # # 17,67 4,14

osa-miR5083 # # 0,19 -2,43

osa-miR6250 8,68 3,12 2,62 1,39

osa-miR6253 # # 0,20 -2,30

osa-miR6255 # # 7,85 2,97

osa-miRf10132-npr 5,73 2,52 # #

osa-miRf10144-npr # # 0,43 -1,23

osa-miRf10193-npr 0,50 -1,01 # #

osa-miRf10194-npr # # 0,48 -1,05

osa-miRf10240-npr # # 0,44 -1,17

osa-miRf10415-npr 0,48 -1,06 # #

osa-miRf10779-npr # # 0,41 -1,30

osa-miRf10806-npr # # 0,15 -2,75

osa-miRf10866-npr 2,39 1,25 # #

osa-miRf10975-npr 0,44 -1,19 # #

osa-miRf11043-npr # # 0,21 -2,23

osa-miRf11202-npr 0,42 -1,26 # #

osa-miRf11216-npr # # 0,50 -1,00

osa-miRf11427-npr # # 0,46 -1,11

osa-miRf11732-npr 0,50 -1,01 0,15 -2,75

osa-miRf11894-npr 0,48 -1,06 # #

osa-miRf11990-npr 4,34 2,12 # #

pab-miR3711 # # 3,93 1,97

peu-miR2910 2,14 1,10 # #

peu-miR2914 # # 0,29 -1,79

pgl-miR159 # # 3,93 1,97

ppe-miR390 # # 0,39 -1,36

ppt-miR160g # # 10,10 3,34

ppt-miR414 2,27 1,18 # #

ppt-miR894 0,47 -1,09 2,86 1,52

pta-miR159a 4,77 2,25 # #

pta-miR159c 0,15 -2,78 0,39 -1,36

ptc-miR159a # # 0,39 -1,36

ptc-miR162b 2,27 1,18 # #

ptc-miR390c # # 0,39 -1,36

ptc-miR396b 0,44 -1,19 # #

ptc-miR396f 0,15 -2,78 5,89 2,56

ptc-miR6478 0,11 -3,18 3,53 1,82

ptc-miRf10034-akr # # 9,22 3,20

ptc-miRf10040-akr # # 0,44 -1,18

ptc-miRf10271-akr # # 10,10 3,34

ptc-miRf10647-akr 0,08 -3,58 0,09 -3,43

ptc-miRf10758-akr # # 0,41 -1,30

ptc-miRf11967-akr # # 0,37 -1,43

pvi-miR444 0,44 -1,19 # #

rco-miR156a 0,44 -1,19 # #

rco-miR156b 0,40 -1,33 # #

rco-miR156c 4,55 2,18 0,39 -1,36

rco-miR156d 0,22 -2,19 0,39 -1,36

rco-miR162 # # 0,39 -1,36

sar-miR159 0,33 -1,59 3,93 1,97

sbi-miR159a 0,50 -1,01 0,13 -2,95

sbi-miR159b 0,44 -1,19 # #

sbi-miR162 0,44 -1,19 # #

sbi-miR164c 0,37 -1,42 3,93 1,97

sbi-miR168 # # 4,28 2,10

sbi-miR396d 0,43 -1,23 13,09 3,71

sbi-miR396e 0,31 -1,67 4,91 2,29

sbi-miR397-3p 0,08 -3,59 # #

sbi-miR398 2,27 1,18 # #

sbi-miR399k 0,44 -1,19 # #

sbi-miR5564a 0,33 -1,59 0,14 -2,88

sbi-miR5564b 0,15 -2,73 2,56 1,36

sbi-miR5565a # # 0,44 -1,17

sbi-miR5565g-3p 0,50 -1,01 5,23 2,39

sbi-miR5568b-3p 0,44 -1,19 # #

sbi-miR5568b-5p 0,44 -1,19 # #

sbi-miR5568f-3p # # 0,39 -1,36

sbi-miR5568g-3p 0,15 -2,78 # #

sbi-miR6217a-3p 3,98 1,99 # #

sbi-miR6217b-5p 0,50 -1,00 0,44 -1,17

sbi-miR6218-3p 0,11 -3,19 0,39 -1,36

sbi-miR6218-3p # # 0,39 -1,36

sbi-miR6219-5p # # 0,44 -1,17

sbi-miR6220-3p 0,25 -2,00 8,83 3,14

sbi-miR6225-3p # # 0,15 -2,75

sbi-miR6225-5p # # 0,22 -2,17

sbi-miR6226-3p # # 8,83 3,14

sbi-miR6232a-3p 0,25 -2,00 # #

sbi-miR6232b-5p 0,22 -2,19 10,10 3,34

sbi-miR6233-5p 2,98 1,58 2,62 1,39

sbi-miR6235-3p 0,13 -3,00 # #

sbi-miR6235-5p 0,50 -1,00 # #

sit-miR121-1-npr # # 0,03 -4,88

sit-miR122-1-npr # # 0,36 -1,49

sit-miR122-2-npr # # 0,03 -5,24

sit-miR148-npr # # 0,39 -1,36

sit-miR156a-1 2,27 1,18 3,93 1,97

sit-miR156a-2 0,50 -1,01 0,19 -2,36

sit-miR156a-4 0,44 -1,19 0,13 -2,95

sit-miR156a-5 2,27 1,18 # #

sit-miR164c 0,22 -2,18 # #

sit-miR16-npr # # 5,89 2,56

sit-miR319-1 0,13 -2,92 3,70 1,89

sit-miR319-1 # # 3,70 1,89

sit-miR319-2 0,25 -2,01 4,42 2,14

sit-miR41-npr 2,27 1,18 # #

sit-miR49-npr 0,25 -2,01 0,19 -2,43

sit-miR50-npr 0,33 -1,59 # #

sit-miR92-npr # # 0,19 -2,36

sly-miR395a 2,17 1,12 28,91 4,85

sly-miR395b # # 9,64 3,27

sly-miR396a-5p 0,44 -1,19 # #

smo-miR159 0,44 -1,19 # #

sof-miR159a 0,20 -2,33 0,13 -2,95

sof-miR159a # # 0,13 -2,95

sof-miR159b # # 7,85 2,97

sof-miR168a # # 14,40 3,85

sof-miR396 2,27 1,18 # #

ssl-miR156 # # 0,39 -1,36

ssp-miR1432 0,33 -1,59 0,13 -2,95

ssp-miR168a # # 4,49 2,17

ssp-miR444a 0,33 -1,59 3,93 1,97

ssp-miR444b 0,25 -2,01 3,93 1,97

ssp-miR444c 2,27 1,18 3,93 1,97

stu-miR479 0,11 -3,19 # #

tae-miR035b_npr 0,44 -1,19 # #

tae-miR041a_npr 0,46 -1,13 # #

tae-miR053a_npr 0,44 -1,19 0,39 -1,36

tae-miR122b_npr 2,17 1,12 19,27 4,27

tae-miR156 0,22 -2,19 # #

tae-miR159a 0,06 -4,00 3,93 1,97

tae-miR159b # # 0,19 -2,36

tae-miR170a_npr # # 0,25 -1,98

tae-miR2018a_npr 2,39 1,25 3,93 1,97

tae-miR2024a_npr 0,25 -2,01 10,60 3,41

tae-miR2028a_1_npr # # 0,08 -3,68

tae-miR2049a_npr # # 0,39 -1,36

tae-miR444c_1_npr 0,22 -2,19 3,93 1,97

tae-miR444c_2_npr 0,11 -3,19 3,93 1,97

tae-miR9773 0,50 -1,00 # #

tcc-miR162 # # 0,39 -1,36

tcc-miR396a # # 10,10 3,34

ttu-miR160b 2,98 1,58 # #

vun-miR162b.1 0,44 -1,19 # #

vun-miR166c*.3 # # 0,39 -1,36

vun-miR395e.1 0,43 -1,23 13,74 3,78

vun-miR408.2 # # 0,39 -1,36

vvi-miR159c 0,44 -1,19 # #

vvi-miR397a # # 0,39 -1,36

zma-miR156a-3p 0,46 -1,13 # #

zma-miR156d-3p 2,17 1,12 # #

zma-miR159a-3p 0,25 -2,01 3,93 1,97

zma-miR159b-3p 3,98 1,99 # #

zma-miR159f-3p # # 5,89 2,56

zma-miR159j-3p 2,73 1,45 3,93 1,97

zma-miR159k-3p 0,15 -2,78 3,93 1,97

zma-miR164b-3p # # 10,60 3,41

zma-miR164e-5p # # 30,29 4,92

zma-miR164f-3p # # 0,39 -1,36

zma-miR166a-5p 0,22 -2,19 0,39 -1,36

zma-miR166d-5p 0,15 -2,78 20,19 4,34

zma-miR167b-3p 0,48 -1,06 36,87 5,20

zma-miR167e-3p 0,46 -1,13 # #

zma-miR167f-3p 0,15 -2,71 # #

zma-miR167g-3p 0,05 -4,26 0,37 -1,43

zma-miR168a-5p # # 8,29 3,05

zma-miR168b-5p # # 9,16 3,20

zma-miR169b-3p 0,44 -1,19 # #

zma-miR169c-3p 0,46 -1,13 # #

zma-miR169m-3p 0,14 -2,85 # #

zma-miR169m-5p 0,22 -2,19 # #

zma-miR169n-5p 0,44 -1,19 0,39 -1,36

zma-miR319c-3p # # 10,60 3,41

zma-miR319d-5p 2,27 1,18 # #

zma-miR393c-5p # # 0,41 -1,30

zma-miR395o-3p 0,22 -2,19 0,39 -1,36

zma-miR396c 0,14 -2,82 4,91 2,29

zma-miR396d 0,05 -4,30 9,64 3,27

zma-miR396g-5p 0,22 -2,19 # #

zma-miR396h 0,44 -1,19 # #

zma-miR398b-3p 0,44 -1,19 # #

zma-miR398b-5p 0,44 -1,19 0,39 -1,36

zma-miR444a # # 30,29 4,92

zma-miR444b 0,28 -1,82 0,39 -1,36

zma-miR528b-5p 0,44 -1,19 # #

zma-miR827-5p 2,27 1,18 # #

^1^FC: Fold change; # Not responsive.
