# Supplementary material for: Microtranscriptome analysis of sugarcane cultivars in response to aluminum stress
Source: PLoS One. 2019 Nov 7;14(11):e0217806. doi: 10.1371/journal.pone.0217806 (PMC6837492; doi:10.1371/journal.pone.0217806)
Supplement: S2 Table — (DOCX) [file pone.0217806.s003.docx]

**S2 Table**. **The primer sequences used in the RT-qPCR validation.**

| miRNA | Specific primer sequence | |
| --- | --- | --- |
|  |  |  |
| miR167 | RT | GTCGTATCCAGTGCAGGGTCCGAGGTATTCGCACTGGATACGACGATGAA |
|  | F | CGCCGAGGTCATGCTGTAGT |
|  |  |  |
| miR168 | RT | GTCGTATCCAGTGCAGGGTCCGAGGTATTCGCACTGGATACGACGTCCCG |
|  | F | CGGCTCGCTTGGTGCAGAT |
|  |  |  |
| miR6253 | RT | GTCGTATCCAGTGCAGGGTCCGAGGTATTCGCACTGGATACGACAACCCA |
|  | F | CGCGAGGAAAGTGGGCAGT |
|  |  |  |
| miR159 | RT | GTCGTATCCAGTGCAGGGTCCGAGGTATTCGCACTGGATACGACCAGAGC |
|  | F | CGGCGGTTTGGATTGAAGGGA |
|  |  |  |
| miR156 | RT | GTCGTATCCAGTGCAGGGTCCGAGGTATTCGCACTGGATACGACTGTGCT |
|  | F | CGGCGGTGACAGAAGAGAGTG |
|  |  |  |
| miR121 | RT | GTCGTATCCAGTGCAGGGTCCGAGGTATTCGCACTGGATACGACCTCATA |
|  | F | GAACGCTGCAGCGCTGAAG |
|  |  |  |
| 18SRNA | F | CTACGTCCCTGCCCTTTGTACA |
|  | R | ACACTTCACCGGACCATTCAA |
|  |  |  |
| Universal | R | CCAGTGCAGGGTCCGAGGTA |
