# Supplementary material for: Microtranscriptome analysis of sugarcane cultivars in response to aluminum stress
Source: PLoS One. 2019 Nov 7;14(11):e0217806. doi: 10.1371/journal.pone.0217806 (PMC6837492; doi:10.1371/journal.pone.0217806)
Supplement: S3 Table — (DOCX) [file pone.0217806.s004.docx]

**S3 Table**. **miRNAs sequences evaluated**.

| miRNA | miRNAs Sequences |
| --- | --- |
| miR167 | AGGUCAUGCUGUAGUUUCAUC |
| miR168 | UCGCUUGGUGCAGAUCGGGAC |
| miR6253 | GAGGAAAGUGGGCAGUUGGGUU |
| miR159 | GAAACGAAUCUUUUAAGUCUAAUU |
| miR156 | UGACAGAAGAGAGUGAGCACA |
| miR121 | CGCUGCAGCGCUGAAGUAUGAG |
