# Supplementary material for: Microtranscriptome analysis of sugarcane cultivars in response to aluminum stress
Source: PLoS One. 2019 Nov 7;14(11):e0217806. doi: 10.1371/journal.pone.0217806 (PMC6837492; doi:10.1371/journal.pone.0217806)
Supplement: S4 Table — Functional annotation of target genes regulated by the most abundant miRNA families differentially expressed. (DOCX) [file pone.0217806.s005.docx]

**S4 Table. Distribution of predicted miRNA targets genes**. Functional annotation of target genes regulated by the most abundant miRNA families differentially expressed.

| **miRNA** | **Potential Targets** | **Target Entry (UniProt)** | **Molecular function** | **Biological process** | **Cellular component** |
| --- | --- | --- | --- | --- | --- |
| 121/122 | *K^+^ uptake permease 10* | Q9SA05 | Potassium ion transmembrane transporter activity (GO:0015079) | Ion transport (GO:0006811) | Plasma membrane (GO:0005886) |
| 156 | *Squamosa promoter-binding protein-like* | Q9FZK0 | DNA binding transcription factor activity (GO:0003700); metal ion binding (GO:0046872) | Regulation of timing of transition from vegetative to reproductive phase (GO:0048510); regulation of transcription (GO:0006355) | Nucleus (GO:0005634) |
| 159 | *MYB domain protein 101* | O80883 | Transcription regulatory region DNA binding (GO:0044212); DNA binding transcription factor activity (GO:0003700) | Gibberellic acid mediated signaling pathway (GO:0009740); positive regulation of abscisic acid-activated signaling pathway (GO:0009789); regulation of leaf morphogenesis (GO:1901371) | Nucleus (GO:0005634) |
|  | *LRR protein* | Q42371 | protein serine/threonine kinase activity (GO:0004674); signaling receptor binding (GO:0005102) | Regulation of cell growth (GO:0001558); regulation of plant organ morphogenesis (GO:1905421); defense response (GO:0006952) | Mitochondrion (GO:0005739); plasma membrane (GO:0005886) |
| 160 | *Auxin response factor* | Q94JM3 | DNA binding transcription factor activity (GO:0003700); sequence-specific DNA binding (GO:0043565) | Auxin-activated signaling pathway (GO:0009734); response to abscisic acid (GO:0009737); negative regulation of cell proliferation (GO:0008285) | Nucleus (GO:0005634) |
| 164 | *NAC domain containing protein* | Q39013 | DNA binding (GO:0003677); DNA binding transcription factor activity (GO:0003700) | negative regulation of abscisic acid-activated signaling pathway (GO:0009788); transcription, DNA-templated (GO:0006351) | Nucleus (GO:0005634) |
| 166 | *Alpha/beta-hydrolases superfamily protein* | F4IZK0 | Lipase activity (GO:0016298) | Lipid metabolic process (GO:0006629) | Cytosol (GO:0005829); plasma membrane (GO:0005886) |
|  | *CBL-interacting protein kinase 1* | Q8RWC9 | ATP binding (GO:0005524); protein serine/threonine kinase activity (GO:0004674) | Intracellular signal transduction (GO:0035556); response to osmotic stress (GO:0006970); response to abscisic acid (GO:0009737) | Nucleus (GO:0005634); plasma membrane (GO:0005886) |
| 167 | *OsWAK receptor-like cytoplasmic kinase* | P0C5E2 | ATP binding (GO:0005524); polysaccharide binding (GO:0030247); protein serine/threonine kinase activity (GO:0004674) | Cell surface receptor signaling pathway (GO:0007166); cellular response to abscisic acid stimulus (GO:0071215); cellular response to water deprivation (GO:0042631) | Plasma membrane (GO:0005886) |
|  | *Copper-transporting ATPase PAA1* | Q9SZC9 | Cation-transporting ATPase activity (GO:0019829); copper chaperone activity (GO:0016531); copper ion transmembrane transporter activity (GO:0005375) | Copper ion homeostasis (GO:0055070); copper ion transmembrane transport (GO:0035434); photosynthetic electron transport chain (GO:0009767) | Chloroplast (GO:0009507); integral component of membrane (GO:0016021); plastid (GO:0009536) |
| 168 | *Tetratricopeptide repeat (TPR)-like superfamily protein* | Q9SZT8 | Endonuclease activity (GO:0004519); zinc ion binding (GO:0008270); RNA binding (GO:0003723) | Chloroplast RNA modification (GO:1900865); mRNA processing (GO:0006397); RNA modification (GO:0009451) | Chloroplast (GO:0009507); intracellular membrane-bounded organelle (GO:0043231) |
|  | *Phosphatase subunit g4-1* | Q9FEE2 | Calcium ion binding (GO:0005509) | Cortical cytoskeleton organization (GO:0030865); preprophase band assembly (GO:0000913); unidimensional cell growth (GO:0009826) | Spindle (GO:0005819); nucleus (GO:0005634); cytoplasm (GO:0005737); phragmoplast (GO:0009524) |
| 169 | *12-oxo-phytodienoic acid reductase 2* | Q8GYB8 | 12-oxophytodienoate reductase activity (GO:0016629); FMN binding (GO:0010181); | Oxidation-reduction process (GO:0055114); oxylipin biosynthetic process (GO:0031408); response to wounding (GO:0009611) | Cytoplasm (GO:0005737) |
| 319 | *MYB domain protein 33* | Q8W1W6 | DNA binding transcription factor activity (GO:0003700); transcription regulatory region DNA binding (GO:0044212); sequence-specific DNA binding (GO:0043565) | Negative regulation of growth (GO:0045926); positive regulation of abscisic acid-activated signaling pathway (GO:0009789); response to ethylene (GO:0009723); response to gibberellin (GO:0009739) | Nucleus (GO:0005634) |
| 390 | *GTP-binding protein obg-like* | Q8L7L0 | GTPase activity (GO:0003924); GTP binding (GO:0005525); magnesium ion binding (GO:0000287) | Chloroplast organization (GO:0009658); thylakoid membrane organization (GO:0010027) | Chloroplast (GO:0009507) |
| 395 | *Sulfate adenylyltransferase* | Q9S7D8 | Adenylylsulfate kinase activity (GO:0004020); ATP binding (GO:0005524); sulfate adenylyltransferase (ATP) activity (GO:0004781) | Hydrogen sulfide biosynthetic process (GO:0070814); sulfate assimilation (GO:0000103) | Chloroplast (GO:0009507); mitochondrion (GO:0005739) |
| 396 | *Growth-regulating factor (GRF)* | Q8L8A6 | ATP binding (GO:0005524); | Leaf development (GO:0048366); regulation of transcription, DNA-templated (GO:0006355) | Nucleus (GO:0005634) |
| 398 | *Copper/zinc superoxide dismutase* | O78310 | Copper ion binding (GO:0005507); superoxide dismutase activity (GO:0004784); zinc ion binding (GO:0008270) | Cellular response to oxidative stress (GO:0034599); response to iron ion (GO:0010039); response to oxidative stress (GO:0006979); cellular response to salt stress (GO:0071472); cellular response to sucrose stimulus (GO:0071329) | Chloroplast (GO:0009507); apoplast (GO:0048046); extracellular space (GO:0005615); thylakoid (GO:0009579) |
| 444 | *MADS-box transcription factor* | P17839 | DNA binding transcription factor activity (GO:0003700); protein dimerization activity (GO:0046983) | Cell differentiation (GO:0030154); positive regulation of transcription by RNA polymerase II (GO:0045944); transcription, DNA-templated (GO:0006351) | Nucleus (GO:0005634) |
| 2128 | *XS domain-containing protein* | Q8VZ79 | RNA binding (GO:0003723) | Response to cadmium ion (GO:0046686); gene silencing by RNA (GO:0031047) | Nucleus - nucleolar ribonuclease P complex (GO:0005655) |
| 5568 | *Phosphofructokinase family protein* | Q9M0F9 | 6-phosphofructokinase activity (GO:0003872); ATP binding (GO:0005524); metal ion binding (GO:0046872) | Fructose 6-phosphate metabolic process (GO:0006002); glycolytic process (GO:0006096) | Cytosol (GO:0005829); plasma membrane (GO:0005886) |
| 6253 | *Nodulin MtN3 family protein* | Q9FM10 | Sugar transmembrane transporter activity (GO:0051119) | Carbohydrate transport (GO:0008643); protein homo-oligomerization (GO:0051260) | Integral component of plasma membrane (GO:0005887) |
